# Treatment of carbon electrodes with Ti_3_C_2_T_*x*_ MXene coating and thermal method for vanadium redox flow batteries: a comparative study

**DOI:** 10.1039/d4ra01380h

**Published:** 2024-04-19

**Authors:** Kavin Teenakul, Sayed Ali Ahmad Alem, Ritambhara Gond, Anupma Thakur, Babak Anasori, Amirreza Khataee

**Affiliations:** a Division of Applied Electrochemistry, Department of Chemical Engineering, KTH Royal Institute of Technology Stockholm SE-100 44 Sweden khat@kth.se; b Montanuniversität Leoben, Institute of Chemistry of Polymeric Materials Otto-Glöckel-Strasse 2 A-8700 Leoben Austria; c Department of Chemistry – Ångström Laboratory Uppsala University Box 538 751 21 Uppsala Sweden; d Department of Mechanical and Energy Engineering, Integrated Nanosystems Development Institute, Indiana University-Purdue University Indianapolis Indianapolis IN 46202 USA; e School of Materials Engineering, Purdue University West Lafayette IN 47907 USA; f School of Mechanical Engineering, Purdue University West Lafayette IN 47907 USA

## Abstract

One of the significant challenges of vanadium redox flow batteries is connected to the negative electrode where the main reaction of V(ii)/V(iii) and the side reaction of hydrogen evolution compete. To address this issue, we used titanium carbide (Ti_3_C_2_T_*x*_) MXene coating *via* drop-casting to introduce oxygen functional groups and metals on the carbon electrode surface. Characterization through scanning electron microscopy and X-ray photoelectron spectroscopy confirmed the even distribution of Ti_3_C_2_T_*x*_ MXene on the electrodes and the presence of titanium and termination groups (–O, –Cl, and –F). The cyclic voltammetry analysis of MXene-coated electrodes showed more sharp electrochemical peaks for the V(ii)/V(iii) reaction than thermal-treated electrodes, even at relatively high scan rates. Notably, a relatively high reaction rate of 5.61 × 10^−4^ cm s^−1^ was achieved for the V(ii)/V(iii) reaction on MXene-coated electrodes, which shows the competitiveness of the method compared to thermal treatment (4.17 × 10^−4^ cm s^−1^). The flow battery tests, at a current density of 130 mA cm^−2^, using MXene-coated electrodes showed pretty stable discharge capacity for over 100 cycles. In addition, the voltage and energy efficiency were significantly higher than those of the system using untreated electrodes. Overall, this work highlights the potential application of MXene coating in carbon electrode treatment for vanadium redox flow batteries due to remarkable electrocatalytic activity and battery performance, providing a competitive method for thermal treatment.

## Abstract 2

Treatment of carbon papers with Ti_3_C_2_T_*x*_ MXene solution for vanadium redox flow batteries.

## Graphical abstract

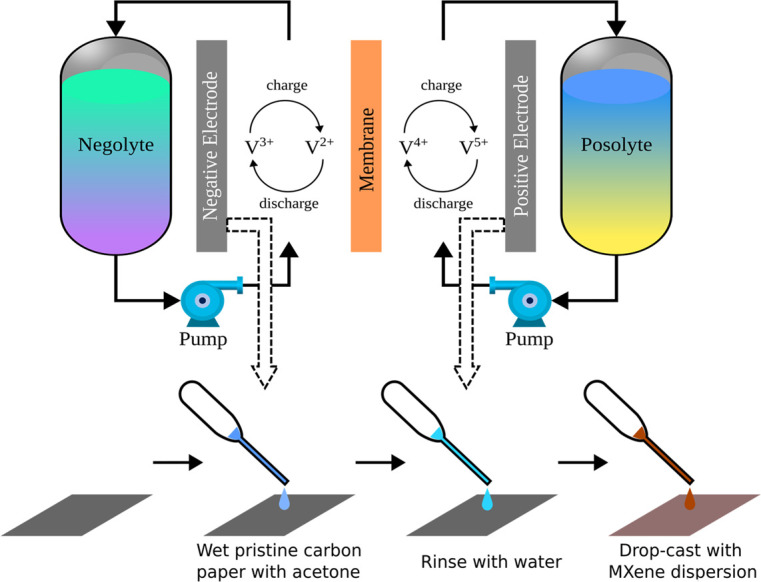

## Introduction

Redox flow batteries (RFBs) are suitable for long-duration and stationary energy storage applications. To date, the main chemistry that has been commercialized for RFBs is based on vanadium.^1–3^ Vanadium redox flow batteries (VRFBs) are attractive due to the four oxidation states of vanadium, which facilitate using vanadium electrolytes on both sides of the cell. Using the same element in both compartments can minimize the electrolyte contamination issue. VRFBs do not suffer from overcharging or a high depth of discharge, and the extremely high chemical stability of vanadium allows the life span of VRFBs to reach 15 000–20 000 cycles.^4^ One of the major challenges of VRFBs is connected to the negative electrode, where the side reaction of hydrogen evolution occurs due to the negative standard potential of the V(ii)/V(iii) reaction. A proper electrode treatment improves the V(ii)/V(iii) reaction while concurrently inhibiting the parasitic hydrogen evolution reaction. The common electrode material used in VRFBs is carbon-based due to high chemical stability, wide operation potential range, and low cost. Carbon felt, paper, and cloth are among the most common forms of carbon-based electrodes used in VRFBs.^5^ Numerous electrode treatment methods have been conducted to improve the vanadium electrochemical reaction, including thermal,^6,7^ chemical (using strong acids),^8–10^ and electrochemical oxidation.^11–13^ The mentioned methods are called intrinsic treatments, which can increase the surface area and the number of active reaction sites by adding oxygen functional groups, which enhance the hydrophilicity and catalytic behavior of the electrode.^5,14–16^ Another strategy is to coat the carbon-based electrodes with metal and metal oxide electrocatalysts to increase the conductivity and catalytic properties of the electrode.^15,17–20^ The same effects have been observed for decorating the electrode with carbon nanomaterials.^15,21–24^ To gain all treatment effects by using one method, MXene coating can be a more straightforward method to enhance the carbon-based electrode electrocatalytic properties and improve the VRFB performance. Two-dimensional transition metal carbides and nitrides, MXenes, where introduced in 2011,^25^ and their unique combination of properties such as high surface area, high electrical conductivity (up to 21 000 S cm^−1^),^26,27^ solution processibility (−40 to −60 mV zeta potential in water),^28^ and rich surface chemistry^29^ have attracted a lot of attention.^30–32^ The electrochemical application of MXene has grown significantly, where MXene has been used in electrochemical energy devices.^33–37^ While both compartments of the battery use vanadium electrolytes, the reaction kinetics of V(ii)/V(iii) and V(iv)/V(v) are different. Previous studies have identified that the catalytic activity of negative electrodes highly depends on electrode treatment, and the kinetics are faster for V(iv)/V(v) than for V(ii)/V(iii).^38,39^ Although MXenes have been used for various applications, they are pretty new in the flow battery field. A. V. Mizrak *et al.* pre-treated a carbon electrode with plasma treatment for two minutes before drop-casting Ti_3_C_2_T_*x*_ MXene onto carbon paper, which enhanced the electrochemical activity of the electrode.^40^ The battery test yielded an energy efficiency of 83% at a current density of 100 mA cm^−2^ with a MXene coating density of 0.1 mg cm^−2^, which they have found to be the optimal coating density. M. Jing *et al.* conducted a heat treatment at 350 °C for 1 hour to enhance the hydrophilicity of the electrode. Subsequently, they immersed carbon felt repeatedly in a MXene dispersion. Their findings revealed that the diffusion coefficient and rate of reaction of the MXene-coated carbon felt increased by two orders of magnitude compared to the pristine carbon felt.^41^ Furthermore, L. Wei *et al.* achieved an energy efficiency of 81.3% at 200 mA cm^−2^ in battery tests by immersing and drying the graphite felt in MXene dispersed in Nafion, where Nafion served as a binder.^42^

To further simplify the process without any pre-treatment or using a binder, in this work, we have developed a simple drop-casting technique that addresses the hydrophobic property of pristine carbon paper by first wetting the electrode followed by drop-casting with Ti_3_C_2_T_*x*_ MXene dispersion. The carbon electrode becomes hydrophilic due to the addition of naturally hydrophilic Ti_3_C_2_T_*x*_ MXene. Our MXene drop-casting method does not require any complex equipment for pre-treatment and can potentially reduce cost and simplify scaling up the process for commercial applications. The electrochemical characterizations and battery tests demonstrated high electrochemical activity and stability toward vanadium electrolytes. Additionally, scanning electron microscopy (SEM) imaging and X-ray photoelectron spectroscopy (XPS) analysis were employed to investigate the characteristics of the electrode surface, explicitly evaluating the exposed surface area and distribution of the MXene coating.

## Experimental

### Materials

Carbon papers (GDL, Sigracet 28AA) and Nafion 212 (N212) membranes were purchased from FuelCellStore. Vanadium(iv) oxide sulfate hydrate, sulfuric acid, and acetone were purchased from Sigma-Aldrich. Hydrofluoric acid (HF, 48–51% solution in water) was purchased from Acros Organics. Lithium chloride (LiCl, 98% grade, Thermo Scientific), and hydrochloric acid (HCl, 12 M) were purchased from Fisher Scientific and used as received. A commercial 1.6 M mixture of V(iii)/V(iv) electrolyte in 2 M H_2_SO_4_ for battery testing was purchased from the GfE company (Gesellschaft für Elektrometallurgie mbH). All chemicals used in cyclic voltammetry and battery testing were used without further purification.

### MXene preparation

To synthesize Ti_3_C_2_T_*x*_ MXene,^26,43^ 1 g of optimized Ti_3_AlC_2_ MAX was first washed using 9 M hydrochloric acid (HCl) for 18 h to remove intermetallic impurities and mixed with an etchant solution (6 : 3 : 1) mixture (by volume) of 12 M HCl, deionized water, and 28.4 M hydrofluoric acid HF before stirring at 400 rpm for 24 h at 35 °C. The etched Ti_3_C_2_T_*x*_ MXene was washed with deionized water *via* repeated centrifugation at 3234 RCF (4–5 cycles with ∼200 ml of deionized water) until the supernatant reached pH ∼ 6. For delamination, the etched multilayered Ti_3_C_2_T_*x*_ MXene sediment was then added to LiCl (typically 50 ml per gram of starting etched powder) solution. The mixture of LiCl and multilayer MXene was then stirred at 400 rpm for 1 h at 65 °C under constant argon gas flow. The mixture was then washed with deionized water *via* centrifugation at 3234 RCF for 5, 10, 15, and 20 minutes. Then, the final mixture was vortexed for 30 minutes followed by centrifugation at 2380 RCF for 30 minutes to ensure the MXene solutions were single-to-few-layered flakes. The final suspension of Ti_3_C_2_T_*x*_ MXene was collected and stored in the freezer at −20 °C until use.

### Electrode preparation

The pristine carbon paper was cut into 5 cm^2^, denoted as an untreated carbon paper electrode. The heat treatment was performed with a muffle furnace (Nabertherm L-051H1RN1T 5/11/B410) at 500 °C for 3 hours in ambient air with a heating rate of 167 °C h^−1^. MXene slurry is stored in the freezer at −15 °C and left to melt at room temperature before use. Ultrapure water (Milli-Q) is mixed with MXene slurry to yield a 5 mg ml^−1^ concentration and sonicated with an ultrasonic cleaning bath (VWR USC 300 T) for 60 minutes.

The pristine carbon paper electrode, possessing hydrophobic properties, can be coated with MXene using a sequential process. The process begins by immersing the electrode in an acetone solution to ensure complete wetting. Afterward, the electrode is rinsed with water to ensure complete removal of acetone. The water-wetted carbon paper is immediately coated with a 5 mg ml^−1^ MXene dispersion using micropipette under normal atmospheric conditions. The wet MXene-coated electrode is dried at 100 °C for 1 hour using a vacuum oven (Heraeus D-6450 Hanau). Both sides of the carbon paper are coated with 0.1 ml, 0.5 ml, and 1 ml of MXene dispersion, resulting in coating densities of 0.1 mg cm^−2^, 0.5 mg cm^−2^, and 1.0 mg cm^−2^ on each side and are labeled MX-0.1, MX-0.5, and MX-1, respectively.

### Characterization of electrode

The surface morphology of the electrodes was analyzed using a Hitachi S-4800 SEM with an accelerating voltage of 10 kV, a working distance of 9400 μm, and an emission current of 10.1 μA without sputtering. The surface composition is analyzed with Kratos AXIS Supra+ instrument XPS using monochromatic Al Kα radiation (1486.6 eV) with calibration using carbon 1s at 284.5 eV. All XPS data was processed using LG4X-V2.^44^

### Cyclic voltammetry

Cyclic voltammetry (CV) was conducted in a three-electrode setup in a beaker. The V(iv) and sulfuric acid solution was prepared by dissolving vanadium(iv) oxide sulfate hydrate and sulfuric acid in water to make a 40 ml mixture of 50 mM V(iv) and a 50 mM sulfuric acid solution. A potentiostat (VersaSTAT 4) was used for CV measurements. Platinum mesh counter electrode and Ag/AgCl reference electrode were used. Electrode samples of 5 cm^2^ are fastened onto a working electrode holder and completely submerged in the solution. The negative side of CV (V(ii)/V(iii)) uses a potential window of 0 V to −0.75 V, and a potential window for the positive side (V(iv)/V(v)) is 0 V to 1.20 V. The scan rates of CV tests for the negative side were 2, 4, 6, 8, and 10 mV s^−1^, while the V(iv)/V(v) scan rates of 3, 5, 10, 20, 30, and 50 mV s^−1^ were used. When the baseline cannot be determined, the semiempirical method suggested by Nicholson is useful to estimate peak anodic current density (*j*_pa_) from peak cathodic current density (*j*_pc_), uncorrected peak current relative to the cathodic peak baseline (*j*_pa,0_), and current density at switching potential, (*j*_sp,0_).^45,46^1
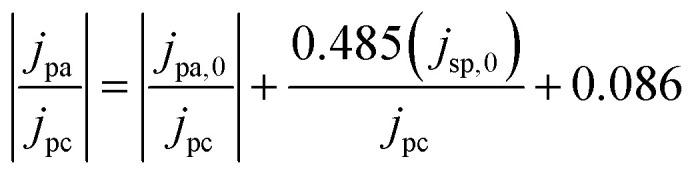


The CV can be further analyzed by calculating the rate of reaction and diffusion coefficient. For single electron transfer processes, when the value of Δ*E*_p_ is greater than 200 mV, the process is considered irreversible.^47^ Therefore, the relationship between peak current density and diffusion coefficient is given by eqn (2), and the relationship with a reaction rate constant (*k*^0^) is given by Randles–Ševčík in eqn (3).^45,46^

2 *j*_p_ = (2.99 × 10^5^)*α*^1/2^*C*_b_*D*^1/2^*v*^1/2^

3
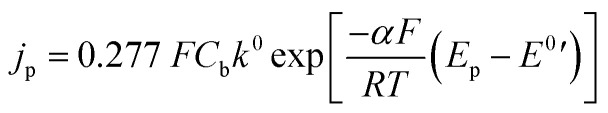


The variables used in the study include *j*_p_, which denotes the peak current density based on the geometrical area (A cm^−2^), *α* denotes the charge–transfer coefficient, *C*_b_ denotes the bulk concentration (mol cm^−3^), *D* denotes the diffusion coefficient (cm^2^ s^−1^), *ν* denotes the scan rate (V s^−1^), *n* denotes the number of electrons, *F* denotes the Faraday constant (C mol^−1^), *E*_p_ denotes the peak potential (V), and *E*^0^′ denotes the formal potential (V). The diffusion coefficient can be calculated by determining the slope when plotting *j*_p_ against the square root of the scan rate and assuming a value of *α* equal to 0.5. The reaction rate constant is determined as the intercept when plotting *j*_p_ against the difference between the peak and formal potential (*E*_p_ − *E*^0^′).

### VRFB single-cell performance

The VRFB tests were conducted using electrolytes containing 1.6 M of V(iii) and V(iv) in 2 M sulfuric acid. The electrochemical cell (Fuel Cell Technologies) was provided with two Poco graphite plates with a serpentine flow field along with two 200 μm Viton gaskets. The electrode configuration is presented in Table 1. In this setup, each side of the cell consisted of a stack comprising two square electrodes, each with a geometric area of 5 cm^2^. The electrolyte was pumped into the electrodes through a single serpentine graphite flow field with an active area corresponding to a geometric area of 5 cm^2^. Nafion 212 was used as the proton exchange membrane. Both the electrolytes were 13 ml, and the external glass bottle containing the negative electrolyte was purged with nitrogen gas to prevent it from oxidizing with atmospheric oxygen. The electrolytes were pumped using a dual-channel peristaltic pump (BT600L, Zhengzhou Mingyi Instrument Equipment Co., Ltd) at a constant volumetric flow rate of 20 ml min^−1^.

**Table 1:** Electrode configurations used in battery performance tests

Negative electrode	Positive electrode
Untreated carbon paper	Heat-treated carbon paper
Heat-treated carbon paper	Heat-treated carbon paper
MX-0.1	Heat-treated carbon paper
MX-0.1	MX-0.1
MX-0.5	MX-0.5

The battery test equipment used in this study was the CT2001A (Landt Instruments). For all the battery charging and discharging setups, constant current charging was set to 650 mA (130 mA cm^−2^) until the voltage reached 1.7 V. This was followed by a resting period of 1 minute. Subsequently, a constant current discharge of 650 mA was applied until the voltage reached 0.8 V, again followed by a resting period of 1 minute. This charge–discharge process was repeated for a total of 100 cycles. It is worth noting that the first cycle was excluded from the analysis due to the unknown state of charge of the initial electrolyte.

For the calculation of efficiencies expressed in percentage, the voltage efficiency (VE), coulombic efficiency (CE), and energy efficiency (EE) of the flow batteries are calculated using eqn (4)–(6). *I*_d_ and *I*_c_ are discharge and charge currents, and *V*_d_ and *V*_c_ are discharge and charge voltages, respectively.4
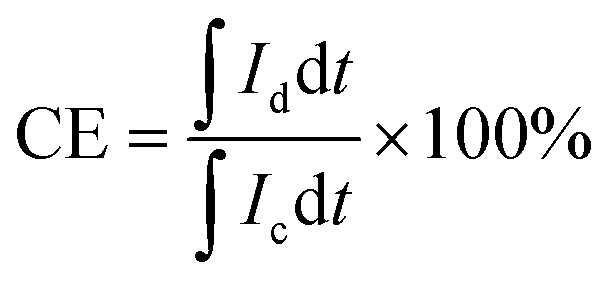
5
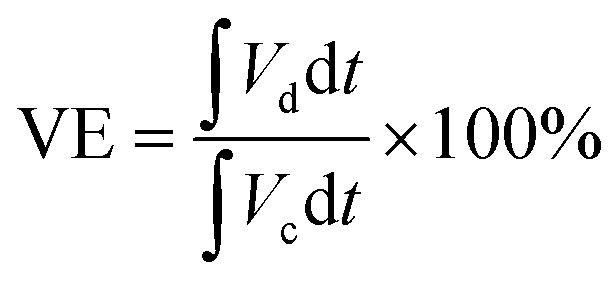
6
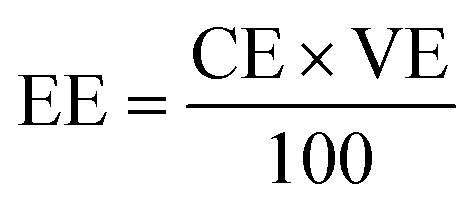


## Results and discussion

**Fig. 1:**
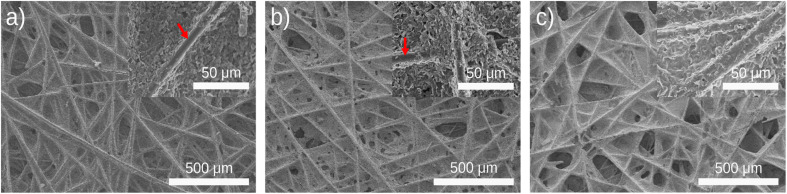
SEM micrographs at 500 μm scale and 50 μm scale (inset) are shown. (a) Untreated carbon paper, (b) MXene-coated untreated carbon paper with a coating density of 0.1 mg cm^−2^, and (c) MXene-coated untreated carbon paper with a coating density of 1.0 mg cm^−2^. The red arrows in the inset in subfigures (a) and (b) point to exposed carbon fibers, while in subfigure (c), a complete coverage of MXene on carbon fiber is shown.

Fig. 1a shows the carbon electrodes before coating of Ti_3_C_2_T_*x*_ MXene, revealing a porous surface structure with binder materials effectively holding the fibers together. The carbon paper electrodes were coated with Ti_3_C_2_T_*x*_ MXene at three different loadings labeled MX-0.1, MX-0.5, and MX-1 (see Methods section). As the loading of Ti_3_C_2_T_*x*_ MXene increased from 0.1 mg cm^−2^ (MX-0.1) to 1 mg cm^−2^ (MX-1), the pores in the electrodes gradually became filled. In Fig. 1b, it is evident that MX-0.1 resulted in a reduction in the carbon paper exposed surfaces and a reduced number of observed pores. By increasing the MXene loading (MX-1), the exposed surfaces were further reduced, and most of the pores were filled with Ti_3_C_2_T_*x*_ MXene. In lower MXene loading (*e.g.*, MX-0.1), some carbon fibers were still exposed (red arrows in figure insets), whereas increasing the Ti_3_C_2_T_*x*_ MXene loading to 1.0 mg cm^−2^ in Fig. 1c resulted in complete coverage of the fibers. A high surface area is essential to reduce activation loss, and a large hydraulic permeability promotes electrolyte transport and reduces pump loss.^48–50^ By increasing the MXene loading from 0.1 mg cm^−2^ to 1 mg cm^−2^, exposed carbon fibers that are electrochemically active sites can be covered. Additionally, the open pores, which are essential for the hydraulic permeability of carbon paper, are mainly covered and closed. As a result of these observations, we expected that lower MXene loadings in MX-0.1 and MX-0.5 are more effective in delivering a redox catalytic effect while maintaining a high active area and electrolyte transport.

**Fig. 2:**
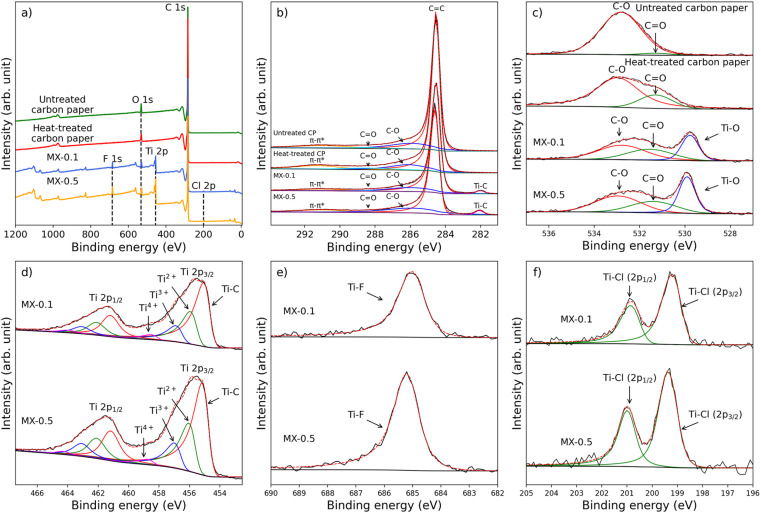
Component peak fits for prepared carbon paper electrodes. (a) Wide XPS spectra survey of untreated carbon paper, heat-treated carbon paper, MX-0.1, and MX-0.5. (b) C 1s (c) oxygen 1s, (d) titanium 2p, (e) fluorine 1s, (f) chlorine 2p.

The XPS analysis of treated carbon paper revealed additional surface functionalities and elements arising from heat treatment and MXene coating. For calibration purposes, the C 1s peak at 284.5 eV is employed, leveraging the high conductivity of carbon paper as a reference point.^51,52^Fig. 2 shows the results of curve fitting for C 1s, O 1s, Ti 2p, F 1s, and Cl 2p. In Fig. 2a, the comprehensive survey spectra exhibit prominent peaks corresponding to carbon (C 1s) at 284.5 eV, oxygen (O 1s) at 532.7 eV, titanium (Ti 2p) at 454.7 eV, fluorine (F 1s) at 684.8 eV, and chlorine (Cl 2p) at 199.0 eV upon the introduction of Ti_2_C_3_T_*x*_ MXene. In Fig. 2b, the C 1s spectrum displays a prominent peak originating from C=C at 284.5 eV. As we move to higher binding energies at 286.0 eV, 288.4 eV, and 291.3 eV, these can be attributed to C–O, C=O, and the π–π* shake-up feature, respectively.^53^ Additionally, the presence of titanium carbide peaks is observed at 282.0 eV with the incorporation of MXene on the MX-0.1 and MX-0.5 electrodes, as represented in Fig. 2b. From Fig. 2a, the quantity of chemisorbed oxygen on the surface of carbon paper remains constant after heat treatment, maintaining an identical atomic percent of oxygen as the untreated carbon paper, both at 13%, assuming an equivalent homogeneous composition. Deconvolution of the C 1s and O 1s spectra reveals the presence of two oxygen functional groups, specifically C–O at 532.9 eV and C=O at 531.3 eV. The percentage of C=O to C–O, as determined from the O 1s curve fitting, exhibits a notable contrast between untreated carbon paper (3.5%) and heat-treated carbon paper (28%). This indicates a preference for the formation of C=O groups during heat treatment while the atomic percentage of oxygen remains constant.

Incorporating Ti_3_C_2_T_*x*_ MXene onto carbon paper in the MX-0.1 and MX-0.5 electrodes introduces two species in O 1s: C=O (531.5 eV) and Ti–O (529.8 eV), resulting in an oxygen atomic percentage of 10% for both MX-0.1 and MX-0.5. From the corroborating findings from prior studies,^53,54^ a deconvolution of the Ti 2p spectrum reveals multiple peaks corresponding to various oxidation states, which are the titanium carbide (Ti–C) peak at 454.7 eV, followed by Ti^2+^ (455.7 eV) arising from Ti–O, Ti^3+^ (456.6 eV) from Ti–F, and Ti^4+^ (458.3 eV) from TiO_2_. Since the MXene coating was conducted at room temperature and the MXene-coated carbon paper was not treated, we do not expect MXene oxidation. However, it is still essential to quantify any MXene oxidation due to MXene ink storage, as any oxidation can reduce MXene high electrical conductivity.^55,56^ Within the Ti 2p spectrum, a minor quantity of TiO_2_, comprising 5% and 3% is found for MX-0.1 and MX-0.5, respectively, in comparison to a substantial presence of Ti–C (53% and 52% for MX-0.1 and MX-0.5, respectively), indicates that oxidation has not progressed far. Furthermore, the presence of peaks in the F 1s and Cl 2p spectra (as seen in Fig. 2c and d) confirms the existence of termination groups, with Ti–F at 685.0 eV and Ti–Cl at 199.1 eV (2p_3/2_) similar to previous reports.^53,54,57^ The oxygen, fluorine, and chlorine atomic percentages are recorded at 10%, 3%, and 1%, respectively, for both MX-0.1 and MX-0.5. Notably, MX-0.5 exhibits a higher Ti atomic percentage of 31% compared to 25% for MX-0.1. In comparison to heat-treatment techniques, the utilization of MXene coating offers distinct advantages due to its inherent hydrophilicity, attributed to its termination groups.^34,58^ By incorporating MXenes as a coating onto pristine carbon paper with the drop casting process proposed in this work, we increase hydrophilicity without requiring additional pre-treatments.

**Fig. 3:**
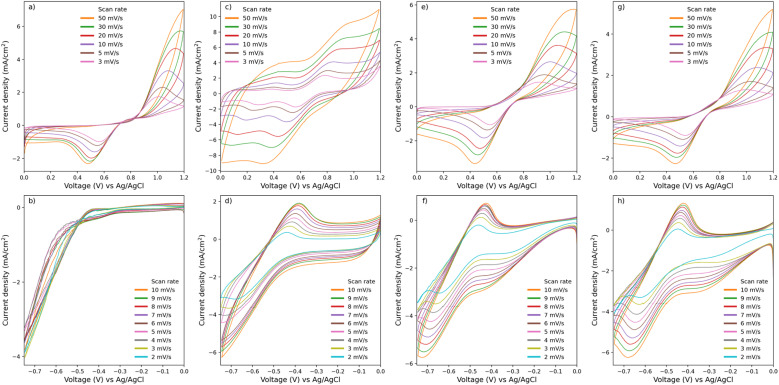
Cyclic voltammograms of various electrodes at different scan rates. Panels (a) and (b) represent the positive and negative sides, respectively, of the untreated carbon paper electrode. Similarly, panels (c) and (d) show the negative and positive sides of the heat-treated carbon paper electrode, respectively. Panels (e) and (f) correspond to the MX-0.1, while panels (g) and (h) depict the MX-0.5 electrode.

Cyclic voltammetry with a three-electrode setup is used to investigate the electrochemical performance of the electrodes. The ratio of anodic peak current to cathodic peak current (*J*_pa_/*J*_pc_) of 1 and the peak-to-peak separation (Δ*E*_p_) of 57 mV indicate an ideal reversible reaction. A derivation of *j*_pa_/*j*_pc_ from 1 and Δ*E*_p_ from 57 mV is considered quasi-reversible.^59^ The CV of electrode samples is shown in 3. The untreated electrode does not show any peak corresponding to the redox of V(ii)/V(iii) species, as seen in Fig. 3b, which indicates that the untreated electrode has no catalytic properties towards the redox of V(ii)/V(iii) species, only a hydrogen evolution peak is observed. On the other hand, untreated carbon paper does show redox peaks for V(iv)/V(v) redox species in Fig. 3a.

With the heat treatment method in Fig. 3c and d, an observable redox peak appears on the negative side of CV for heat-treated carbon paper electrodes, and with MXene-coated carbon paper electrodes, a catalytic property towards V(ii)/V(iii) has transformed from no activity to a noticeable enhancement. The cathodic peak of heat-treated carbon paper on the negative side shifts to the lower potential until the peak is not observed beyond the scan rate of 4 mV s^−1^. In Fig. 3c, a large non-faradaic current is observed in heat-treated carbon paper on the positive side compared to untreated carbon paper, MX-0.1, and MX-0.5, which might be owing to the higher electrolyte wetting on the surface of heat-treated carbon paper compared to untreated, MX-0.1, and MX-0.5, leading to a larger electrochemically active surface area and capacitance.^60^

**Fig. 4:**
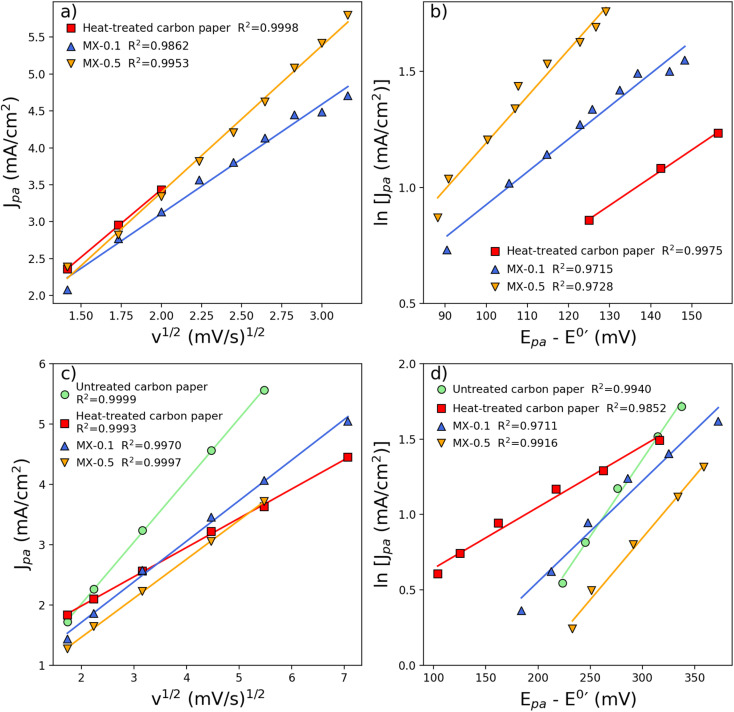
The relationship between peak current density and the square root of the scan rate (a and c) demonstrates a linear correlation. Additionally, a linear relationship exists between the natural logarithm of peak current density and peak separation. Subplots (a and b) are for the negative side, while subplots (c and d) pertain to the positive side.

The kinetics of the electrodes towards vanadium species were investigated by analyzing the relationship between anodic peak current, scan rate (*v*), and peak separation, as illustrated in Fig. 4. To determine the diffusion coefficient, *J*_pa_ was plotted against the square root of the scan rate (*v*^1/2^), resulting in a linear curve. The slope of this curve (Fig. 4a and c), was used in eqn (2). The linearity observed, along with a peak separation greater than 200 mV, indicates an irreversible electrode process. Additionally, to determine the *k*^0^, the natural logarithm of *J*_pa_ was plotted against (*E*_p_ − *E*^0^′), yielding intercepts. The results of the diffusion coefficient and reaction rate constant from CV are summarized in Table 2.

**Table 2:** Diffusion coefficient and reaction rate constant from CV

Electrodes	V(ii)/V(iii)		V(iv)/V(v)	
	*D* (cm^2^ s^−1^)	*k* ^0^ (cm s^−1^)	*D* (cm^2^ s^−1^)	*k* ^0^ (cm s^−1^)
Untreated carbon paper	—	—	9.40 × 10^−6^	1.67 × 10^−4^
Heat-treated carbon paper	3.00 × 10^−5^	4.85 × 10^−4^	2.59 × 10^−6^	1.11 × 10^−3^
MX-0.1	1.96 × 10^−5^	5.61 × 10^−4^	4.05 × 10^−6^	4.17 × 10^−4^
MX-0.5	3.53 × 10^−5^	4.06 × 10^−4^	3.74 × 10^−6^	1.81 × 10^−4^

In contrast to the negative electrode reaction, on the positive side, every electrode except heat-treated carbon paper electrode shows the same order of magnitude for the diffusion coefficient as shown in Table 2. Only heat-treated carbon paper shows an increase by one order of magnitude for the rate of reaction. Only a slight increase in the rate of reaction is observed in MX-0.1 compared to untreated carbon paper. On the other hand, the addition of MXene shows an improvement in diffusion coefficient and rate of reaction compared to untreated carbon paper electrode for the reaction of V(ii)/V(iii), untreated carbon paper electrode catalytic activity towards V(ii)/V(iii) is not observable as seen in Fig. 3b. These results suggest that the coating of MXene does not improve catalytic activity towards V(iv)/V(v). Similarly, for heat treatment, C. Choi *et al.*^39^ and I. Derr *et al.*^61^ conclude that the electrode reaction with V(iv)/V(v) is an outer-sphere mechanism that shows less dependence of on heat treatment than V(ii)/V(iii). The diffusion coefficient and rate of reaction for MX-0.1 and MX-0.5 rival those of a heat-treated electrode. From the XPS results, despite the abundance of carbonyl groups (C–O) on the surface of untreated carbon paper, electrochemical activity towards V(ii)/V(iii) species is not observed in the CV experiments. The high electrochemical activity towards V(ii)/V(iii) species for heat-treated carbon paper suggests that carbonyl groups (C=O) serve as active catalysts for V(ii)/V(iii) species and not carboxyl groups (C–O), despite the equal quantity of total oxygen content between untreated carbon paper and heat-treated carbon paper. These findings align with the work of I. Derr *et al.*, who proposed that C–O groups inhibit activity, while C=O groups catalyze an inner-sphere mechanism on the negative side of the electrochemical cell.^61^

**Fig. 5:**
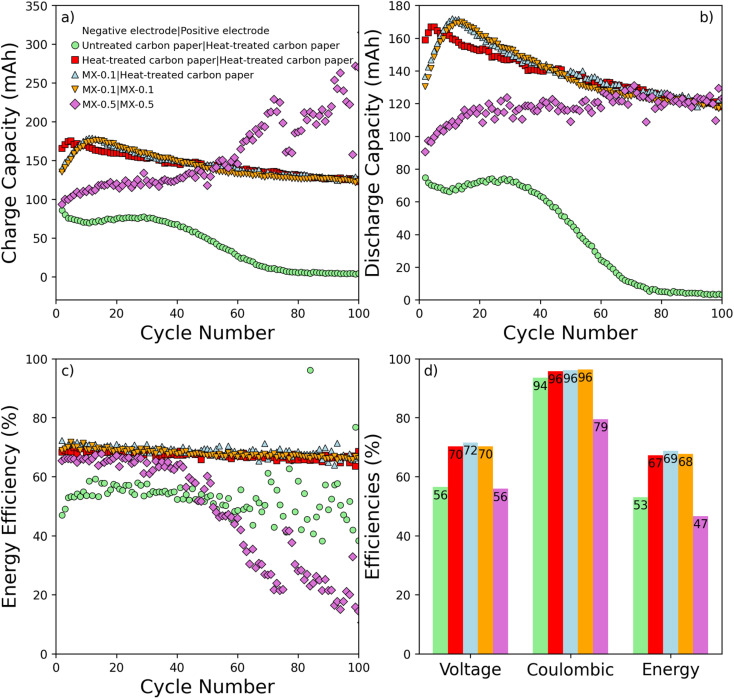
Battery performance tests were conducted with various electrode configurations over 100 cycles at a current density of 130 mA cm^−2^. The electrolyte consisted of 1.6 M vanadium and 2 M sulfuric acid, with a constant volumetric flow rate of 20 ml min^−1^ maintained throughout all tests. (a) Charge capacities, (b) discharge capacity, (c) energy efficiency, and (d) average energy efficiency.

However, it is important to note that there is no consensus on the detailed mechanism or the specific functional groups responsible for these effects.^38^ Cyclic voltammetry for MX-1 is not presented here, but the underlying reasons for this will be clarified in the subsequent section on battery testing, where performance issues specific to the high MXene coating density of MX-0.5 are discussed in detail. The capacities and efficiencies of VRFB with different electrode configurations are tested, as shown in Fig. 5. The electrode configurations heat-treated carbon paper|heat-treated carbon paper, MX-0.1|heat-treated carbon paper, and, MX-0.1|MX-0.1 are shown to have similar performance in terms of capacities and efficiencies. The result of VRFB tests on this electrode configuration is shown in Fig. 5. The charge capacity and discharge capacity are shown in Fig. 5a and b. The configuration heat-treated carbon paper|heat-treated carbon paper, MX-0.1|heat-treated carbon paper, and MX-0.1|MX-0.1 reach discharge capacity of 118 mA h, 120 mA h, and 118 mA h after 100 cycles, respectively. Untreated carbon paper|heat-treated carbon paper configuration experiences a sharp decline in discharge capacity; only 3.4 mA h of discharge capacity is available after 100 cycles. The configuration MX-0.5|MX-0.5 exhibits increasing discharge capacity, which reaches 123 mA h.

From the discharge capacity, electrolyte utilization is an important metric to evaluate energy density, which is important to reduce the total volume of the electrolyte. The electrolyte utilization is defined as a ratio of measured discharge capacity to theoretical capacity (557 mA h).^62^ Electrolyte utilization of untreated carbon paper|heat-treated carbon paper, heat-treated carbon paper|heat-treated carbon paper, MX-0.1|heat-treated carbon paper, MX-0.1|MX-0.1, and MX-0.5|MX-0.5 after 100 cycles are 0.61%, 21.2%, 21.5%, 21.2%, and 22.0%, respectively. It should be mentioned that the discharge capacity shows an increase in the first couple of cycles due to using Nafion membrane and is explained by the fast non-equilibrium electrolyte crossover in the initial cycles.^63,64^

The EE is shown in Fig. 5c, in which untreated carbon paper|heat-treated carbon paper and MX-0.5|MX-0.5 show poor EE over a 100-cycles, while heat-treated carbon paper|heat-treated carbon paper, MX-0.1|heat-treated carbon paper, and MX-0.1|MX-0.1 show minimal degradation in EE. The average energy efficiencies are depicted in Fig. 5d. Overall, the average EE of MX-0.1|heat-treated carbon paper is measured at 69%, slightly surpassing the efficiency of MX-0.1|MX-0.1 at 68%. These values represent an improvement compared to heat-treated carbon paper|heat-treated carbon paper, which achieved an average EE of 67%. Conversely, untreated carbon paper|heat-treated carbon paper demonstrated a significantly poorer EE, measuring only 53%. Remarkably, the tests involving VRFB electrodes with an increased MXene loading up to 0.5 mg cm^−2^ did not exhibit EE enhancements, as observed in MX-0.5|MX-0.5. Rather, a large drop in average VE and CE is observed. We observed the lowest average CE of 79% in MX-0.5|MX-0.5. However, the CV results indicated that MX-0.5 exhibited a significantly high diffusion coefficient and rate of reaction, as shown in Table 2. These findings suggest that MX-0.5 possesses favorable kinetic properties for vanadium redox species. However, the battery test results revealed that the CE for the MX-0.5 only reached 79% and the VE to 56%. The decreased CE can be attributed to the reduced active surface area and hydraulic permeability resulting from the high loading of MXene in MX-0.5. The higher MXene loading likely hinders effective transport by increasing flow resistance and non-uniform distribution inside the electrode, causing a dead zone in the cell compartment,^65,66^ leading to lower average CE and average VE. Thus, a battery test with MX-1 with MXene loading of 1 mg cm^−2^, which has a higher MXene loading than MX-0.5, was not conducted.

Capacity retention is defined as a ratio of discharge capacity at the 100th cycle to initial discharge.^67^ The capacity retention of untreated carbon paper|heat-treated carbon paper is abysmal at 4.12%, resulting from the poor catalytic activity toward V(ii)/V(iii). The cell setups heat-treated carbon paper|heat-treated carbon paper, MX-0.1|heat-treated carbon paper, and MX-0.1|MX-0.1 show much higher capacity retention of 70.8%, 69.9%, and 69.2%, respectively. Despite the rapid increase in capacity retention, reaching 144% after 100 cycles for MX-0.5|MX-0.5, a phenomenon tentatively attributed to the increased wetting of the carbon paper, charge capacity rapidly increases as well, which reduces CE, and therefore, the average EE of the VRFB is drastically reduced to 47%. While MX-0.1 and MX-0.5 show similar diffusion coefficients and rates of reaction in CV, the battery test shows that MX-0.5 is not suitable for VRFB because the energy efficiency significantly drops after 40 cycles. With MX-0.1, the energy efficiency and capacity are competitive with heat-treated carbon paper, which is at 67%. MX-0.1 is employed on the anodic side and on both the anodic and cathodic sides, which exhibit an energy efficiency of 69% and 68%, respectively. Overall, MX-0.1 is a suitable candidate for heat-treated carbon paper for a full-cell VRFB, with the possibility of optimizing the coating density so that the decrease in the active surface area does not hinder the VRFB's performance, as observed in MX-0.5. The observed improvement in electrochemical performance, particularly in the case of MX-0.1, can be attributed to a combination of factors associated with the unique properties of Ti_3_C_2_T_*x*_ MXene. The presence of oxygen, chlorine, and fluorine functional groups, as evidenced by XPS analysis, contributes to the modification of the electrode surface, creating active sites that promote the catalytic activity of V(ii)/V(iii) redox species. Additionally, the controlled MXene loading in MX-0.1 strikes a balance, ensuring the preservation of a high surface area while preventing excessive pore filling, which is crucial for maintaining accessibility to electrochemically active sites and facilitating electrolyte transport. Thus, the combination of surface functional groups and maintaining electrode surface porous structure makes MX-0.1 a favorable choice for enhancing the overall performance of the VRFB. Additionally, in instances where the MXene coating on carbon paper exhibits non-uniformity, areas lacking the MXene coating tend to mimic the behavior of untreated carbon paper. Therefore, the electrode region of insufficient MXene coating will not have catalytic activity towards V(ii) and V(iii) as evident from the cyclic voltammogram in Fig. 3b. Thus, ensuring a uniform coating becomes crucial.

## Conclusions

In this work, we have successfully demonstrated a straightforward drop-casting technique for coating MXene suspension onto untreated carbon electrodes. This method eliminates the need for heat treatment, binders, or any other pre-treatment steps. Our findings indicate that this technique provides a simple and viable alternative to modifying electrodes, yielding electrochemical performance on par with the standard heat-treatment approach. Analysis of SEM images revealed that the MXene particles exhibited minimal agglomeration on the electrode surface and evenly covered the carbon paper. However, increasing the MXene loading could reduce the carbon paper performance. The MX-0.1 with a MXene coating density of 0.1 mg cm^−2^ effectively preserved the exposed surface area of the carbon paper, while higher loadings, *i.e.*, MX-0.5 and MX-1.0 reduced the exposed surface area as the MXene flakes filled the pores. Therefore, further investigation into optimal coating density is needed to improve the battery's performance. Introducing Ti_3_C_2_T_*x*_ MXene led to the detection of various termination groups, including oxygen, chlorine, and fluorine, on the electrode surface. Using the MX-0.1 for both negative and positive electrodes exhibited competitive battery performance in terms of capacity at 118 mA h, which is equivalent to that of a heat-treated carbon paper electrode, as well as an EE of 68% compared to using all heat-treated carbon paper electrodes with an EE of 67%. In conclusion, the drop-casting technique for coating Ti_3_C_2_T_*x*_ MXene onto carbon paper presents a compelling alternative to the conventional heat treatment method. This simple modification improved electrochemical performance and eliminated complex pre-treatment steps. The findings from this study open new possibilities for simple electrode modification techniques in various applications. For future research, it is crucial to thoroughly examine the long-term battery cycling, explore different current densities, and further optimize the coating density of MXene-coated carbon electrodes to assess stability and catalytic activity. Additionally, gaining insights into how MXene interacts with carbon paper and the vanadium electrolyte is essential for advancing our understanding in this area.

## Author contributions

Kavin Teenakul: conceptualization, methodology, validation, investigation, writing – original draft, writing – review & editing. Sayed Ali Ahmad Alem: conceptualization, methodology, review & editing, investigation. Ritambhara Gond: writing – review & editing, investigation. Anupma Thakur: validation, writing – review & editing, advising. Babak Anasori: writing – review & editing, advising. Amirreza Khataee: conceptualization, writing – review & editing, advising.

## Conflicts of interest

The authors declare that they have no known competing financial interests or personal relationships that could have appeared to influence the work reported in this paper.

## Supplementary Material
